# Influence of working conditions and salary on agency work for intermediate and intensive care units: Part 2: Partial results of a nationwide survey

**DOI:** 10.1007/s00063-022-00969-7

**Published:** 2022-11-24

**Authors:** C. Hermes, U. Gaidys, K. Blanck-Köster, E. Rost, C. Petersen-Ewert

**Affiliations:** 1 Hochschule für Angewandte Wissenschaften Hamburg (HAW Hamburg), Alexanderstr. 1, 20099 Hamburg, Germany; 2 IU Internationale Hochschule GmbH, IU University of Applied Sciences, Juri-Gagarin-Ring 152, 99084 Erfurt, Germany; 3 Fakultät Wirtschaft und Soziales—Department Pflege and Management, Hochschule für angewandte Wissenschaften Hamburg, Hamburg, Germany

**Keywords:** Agency workers, Permanent employees, Temporary work, Quality of health care, Critical care, Zeitarbeiter, Festangestellte, Zeitarbeit, Qualität der Gesundheitsversorgung, Intensivpflege

## Abstract

**Background:**

Agency work in nursing is used as a form of labor to counter vacant staff positions in hospitals. Both hospital owners and nurses view this critically for different reasons.

**Aim:**

The aim of this study was to assess what personal net income nurses in German intensive care units and intermediate care units consider “fair and sufficient” for their work (addressed in Part 1 of the survey) and what influence—aside from the salary—the working conditions have on the willingness to change to temporary work or back to a permanent position.

**Methods:**

From September to October 2020, an anonymous online survey was conducted among nurses of intermediate care units, intensive care units, and special care units in German-speaking countries. Descriptive statistics were used for the analysis.

**Result:**

Of 1203 participants, 86% (*n* = 1036) could be evaluated. None of the job satisfaction factors queried received four or five stars (maximum five stars) from those participating in the survey. The most unsatisfied group proved to be regularly employed nurses with an additional part-time job. Key job satisfaction factors differed markedly between the groups, with regular employees favoring consistency and stability. Agency workers prefer gaining experience in a broader range of tasks. Unreliable duty rosters and poor nurse to patient ratios were common points of criticism.

**Conclusion:**

For job satisfaction, making nurses feel appreciated and respected is essential. This includes a guaranteed nurse to patient ratio and reliable duty rosters that also include tasks outside direct patient care. In order for nurses to leave agency work, it is necessary to take into account the differences in interests in terms of the focus of activity.

## Abstract (de)

**Hintergrund:**

Leiharbeit in der Pflege wird als Arbeitsform genutzt, um offenen Personalstellen in Kliniken zu besetzen. Sowohl Krankenhausträger als auch Pflegekräfte sehen dies aus unterschiedlichen Gründen kritisch.

**Ziel:**

Zweck dieser Untersuchung war es herauszufinden, welches persönliches Nettoeinkommen Pflegekräfte von deutschen Intensiv- und Intermediate-Care-Stationen als „gerecht und ausreichend“ für ihre Tätigkeit empfinden (Teil 1 der Umfrage) und welchen Einfluss neben dem Gehalt die Arbeitsbedingungen auf die Wechselwilligkeit in die Leih‑/Zeitarbeit bzw. wieder zurück in eine Festanstellung hat.

**Methode:**

Von September bis Oktober 2020 wurde eine anonymisierte Onlineumfrage unter Pflegenden von Intermediate-Care-Stationen, Intensivstationen und Funktionsbereichen im deutschsprachigen Raum durchgeführt. Die Auswertung erfolgte mittels deskriptiver Statistik.

**Ergebnis:**

Von 1203 Teilnehmer_innen konnten 1036 (86 %) von in Deutschland Arbeitenden ausgewertet werden. Die Zufriedenheit wurde in allen Aspekten mit maximal 3 von 5 Punkten bewertet. Die unzufriedenste Gruppe bildeten die Festangestellten mit zusätzlichem Nebenjob. Die Schlüsselkriterien zur Zufriedenheit unterschieden sich zwischen den Gruppen – so zogen Festangestellte Stetigkeit und Stabilität bei der Tätigkeit vor. Leiharbeiter dagegen waren an einer möglichst breiten Erfahrung interessiert. Unzuverlässige Dienstpläne und schlechte Betreuungsschlüssel wurden allgemein kritisiert.

**Schlussfolgerung:**

Ein wertschätzender, respektvoller Umgang ist für die berufliche Zufriedenheit des Pflegepersonals essenziell. Dies schließt einen garantierten Betreuungsschlüssel sowie verlässliche Dienstpläne, die auch patientenferne Tätigkeiten berücksichtigen, ein. Ist es das Ziel, Pflegepersonal zur Rückkehr aus der Leiharbeit in die Festanstellung zu bewegen, ist eine Berücksichtigung der unterschiedlichen angestrebten Tätigkeitsschwerpunkte notwendig.

While the number of hospitals in Germany has decreased from 2400 to 1942 in the 30 years preceding the current coronavirus disease 2019 (COVID-19) pandemic, the number of cases treated in them has increased by 25% over the same time period [11]. Even without the pandemic, this concentration of workload was expected to further increase as demographic trends lead to an increase in the elderly population and consequently the number of those requiring geriatric and medical care, with a concurrent reduction in the working population [16].

This current and expected further rise in demand [9] is contrasted by a significant reduction in hospital personnel, especially in nursing and care, mediated by pressure to cut costs [13]. This puts into perspective the nominally high rate of intermediate and intensive care beds per 100,000 inhabitants in Germany of about 30 [12]. While indeed the number of beds has increased, the number of nurses not only has not increased correspondingly, but actually decreased [7]. Consequently, conditions in intensive care units (ICUs) more and more frequently prohibit the full number of beds or treatment units being made available [8]. The hospitals often resort to agency workers [AW] and other methods to make their roster more flexible and fill gaps in personnel [10].

This increase in workload, however, leads increasingly to burn-out, medical leave and, more and more frequently, the decision to leave the sector altogether, thereby only exacerbating the problem in a vicious cycle [15], which at the same time makes recruiting sufficient personnel increasingly difficult. While internationally, the use of agency work to plug gaps has been shown to have been able to stabilize labor markets and salaries [6], more recently, at least for Germany, the job satisfaction aspects have increasingly endangered good patient care [9].

Agency work is sometimes seen as being able to pick and choose, selecting better working conditions (more attractive shifts, etc.) while enjoying more favorable compensation structures. This could provide a pull factor away from direct hospital employment, exacerbating the situation of the hospitals. Consequently, German federal authorities have been discussing a potential ban of agency work in this environment.

It is therefore important to understand the effects of agency work on both patient care and the working environment, as well as the factors affecting job satisfaction and the choice to move from one form of employment to the other.

An initial literature review demonstrated that while no significant effects in outcome could be found, a difference in perspective on subjective outcomes was certainly observed [5]. A follow-up survey was conducted to directly measure work conditions and satisfaction. While a previous manuscript in this journal, in German, evaluated aspects of compensation, here, the goal is to evaluate responses on nonmonetary aspects of job satisfaction.

## Design and methodology

As described previously, an anonymized survey among nurses in intensive care and other specialized units in German-language countries was conducted from September to October 2020 using “Survey Monkey”. (This section is a translation of the corresponding section in [4]).

The design followed established recommendations for online surveys [3] as a cross-sectional study with ex post facto analysis. The basic statistical population of “nurses in intensive and intermediate care units” in Germany has not been reliably defined and measured.

The call for participation in the survey was published in three national intensive care nursing journals (*PflegenIntensiv, Intensiv, Medizinische Klinik—Intensiv und Notfallmedizin*) as well as their online portals. In addition, the call was distributed in the entire German-speaking region through professional associations, their mailing lists, social media channels (Twitter, Facebook, WhatsApp) and published on some of the home pages of those associations. These supporting associations were Deutsche Gesellschaft für internistische Intensiv- und Notfallmedizin e. V. [DGIIN] (German Society for Internal Intensive Care Medicine and Emergency Medicine), Deutsche Gesellschaft für Fachkrankenpflege und Funktionsdienste e. V. [DGF] (German Society for Specialized Nursing and Functional Services), Schweizerische Gesellschaft für Intensivmedizin [SGI] (Swiss Society for Intensive Care Medicine), and Österreichische Gesellschaft für Allgemeine Intensivmedizin und Notfallmedizin [ÖGAIN] (Austrian Society for General Intensive Care Medicine and Emergency Medicine). In addition, individual hospitals in the German-speaking areas of Belgium, Luxemburg, Liechtenstein, and Italy (Bozen/Bolzano) were directly contacted.

The invitation contained a brief description of the goal, a link to the associated website and a request to further distribute the invitation to maximize reach. Because of the latter measure, an accurate response rate is not calculable. Finally, the authors and some colleagues directly approached potential participants at conferences and professional education events.

Participants could stop or suspend answering the survey at any time. There was no incentive or compensation offered for participation, which was completely voluntary. This was stressed in the invitation and explicitly confirmed by the participants as part of the questionnaire.

To ensure that ability to resume and to ensure all devices could only participate once, the IP address of participants was temporarily recorded in accordance with the General Data Protection Regulation (GDPR). As such, at least temporarily, some conclusions at least on the institution from which the participant was answering the survey—and potentially the participant—was theoretically conceivable. The information was verified by the first author and a statistician and no abnormalities or patterns that would allow such conclusions were noted.

A review request with the local ethics committee was not filed, as this was deemed unnecessary both by a member of the ethics committee of the protestant hospitals Bielefeld as well as the principal investigator at the University of Applied Sciences Hamburg—participation was voluntary and the participant population was not deemed vulnerable or dependent on the investigating team.

Reminders were distributed via email and social media channels and the survey was closed after less than five responses were received on several consecutive days.

A previous survey for the State Nursing Board of Lower Saxony, conducted by the German Institute for Applied Nursing Research (Deutsches Institut für angewandte Pflegeforschung e. V. [DIP]) served as a basis for the questionnaire. To validate the design, the questionnaire was used in a pretest among under equal numbers of nurses in regular employment with and without side jobs on the one hand and agency workers on the other. The results of this pretest were not included in the final analysis but used to fine-tune questions and formulation. The final questionnaire consisted of several partially independent, partially sequential modules with a total of 84 questions, including some open questions with free text fields (full questionnaire available on request from the first author). The questionnaire included logical routing between modules based on previous answers. This allowed to ensure that employees with or without side jobs and agency workers each only saw questions relevant and specific for their situation. Similarly, the questionnaire distinguished between classical employees who had in the past already worked together with agency workers and those who had not. While the participants could jump back and forth between the individual sections offered to them, they could not view the entirety of the questionnaire. To avoid question order effects [1], automated randomization of questions was used. The question types used ranged from dichotomous questions over multiple choice questions, including with multiple answers, to free text questions and evaluation scales from 0–100. For scaling, modified Likert scales and in some cases scales as semantic differentials were used.

The evaluation was conducted using the statistical software JASP‑0.14® under Open-Source license. Operationalization and categorization were conducted via allocation to various categories, wherever unequivocally possible, or otherwise to “miscellaneous”. The category “I don’t know” was consciously omitted to insist on an explicit decision by the participants. Free text options were usually used for supplementary comments. To avoid a selection bias due to preconceptions on the part of the investigator, operationalization and categorization of the variables were conducted by an independent third party experienced in social research before the eventual evaluation was conducted.

Univariate and bivariate measurands were assessed using descriptive statistics. For variables with normal distribution, arithmetic mean (AM) and standard deviation (SD) are listed. For nonnormally distributed variables, median (MD) and interquartile range (IQR) are provided. Frequency is listed in absolute and relative (%) terms. For two groups, significance is assessed by chi squared (Χ^2^) test, and for more than two using analysis of variance (ANOVA). In the latter case, a post hoc analysis was conducted. Where the requirements for parametric approaches were not met, Kruskall–Wallis tests were done.

## Results

This section addresses the results on job satisfaction and expectations. The results on monetary compensation, being more dependent on factors specific to Germany, can be found in a separate, German-language publication [4].

A total of 1203 participants accessed the questionnaire. The mandatory minimum information was provided by 86% of those (*n* = 1036). The majority of participants was female, resident in Germany, and with a hospital as main employer.

**Fig. 1:**
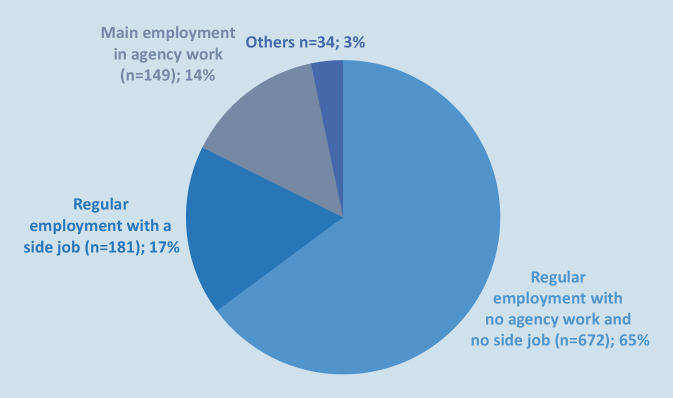
Composition of participants

In all, 65% (*n* = 672) reported being in regular employment without side job. A further 17% (*n* = 181) of participants reported being in regular employment, but also having a side job. Of these, 50 reported a side job in agency work, 131 reported a side job without agency work. A main employment in agency work was reported by 14% of participants (*n* = 149). Furthermore, 3% (*n* = 34) reported being employed in some other fashion (Fig. 1).

Of the 199 participants (19%) who answered that they were active in agency work either as their main form of employment or as a side job, 154 (77%) answered the questions specific for this type of employment. Of these, 97 (63%) stated that they were working in this type of employment for no longer than 3 years. Primary deployment location was the intensive care unit (78%, *n* = 120), while for 21% (*n* = 33), it was a different monitored area. For a plurality of these participants (45%, *n* = 69), these locations were at major hospitals with more than 600 beds.

These participants in agency work were asked to provide information on their work environment. In all, 72 (47%) stated that they did not receive any onboarding at the hospitals they were deployed to. The majority of these stated that they were nonetheless expected by the hospitals to handle equipment without being briefed in its use as provided for by the medical products law.

Agency workers stated they were only rarely transferred to regular wards (*n* = 128, 90%). Most also were not required to participate in patient transport (*n* = 115, 80%), or involved in acute situations like shock trauma room duty, or resuscitation (*n* = 123, 85%). In addition, 96 (67%) noted that they were not expected to participate in team meetings or other official meetings. Regarding a potential move back into regular employment, 116 (76%) noted it would be important or very important for them even with a singular employer to be able to gain experience in a variety of aspects of nursing work. Another requirement for moving back to regular employment listed was also cited as a demand by regular employees: a fixed, nonnegotiable nurse/patient ration through all shifts, on all days, so that workload was both anticipable and manageable.

To verify whether regular employees with and without a side job could be analyzed as a single group for questions not specific to having a side job, a preliminary analysis was done, which found no significant differences in terms of age distribution, gender, or job experience. Of the 672 participants in regular employment without side job, 607 (90%) answered the specific questions for this constellation. Of these, by far the most worked in a hospital environment (*n* = 561, 84%) and 153 (23%) have worked at their current employer for more than 20 years. The largest group (37%, *n* = 249) worked in a hospital with more than 800 beds. More than two thirds (*n* = 411) reported having a full position with 38.5 to 44 h per week. Of the 181 regular employees with side job, 178 (98%) answered further questions.

In terms of job satisfaction, slight differences were observed between employees with and without a side job. Of those without a side job, most (*n* = 381, 63%) could not conceive ever working via an agency. However, only 20% (*n* = 120) of them were actually interested in taking on a side job.

**Fig. 2:**
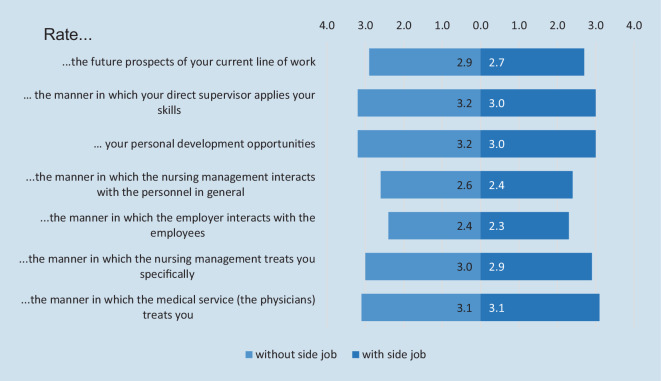
Satisfaction with and without a side job (5-point scale, 5 = very good)

Asked to grade their job experience “with up to 5 stars”, i.e., on a five-point Likert scale, none of the factors queried received four or five stars (Fig. 2). The eight questions assessing various factors of job satisfaction were posed to both groups of regular employees with and without side job, and the latter further stratified based on whether they had a side job in agency work. Analysis of variance showed no marked difference, though the regular employees with a side job in agency work had the lowest relative satisfaction for several factors.

**Fig. 3:**
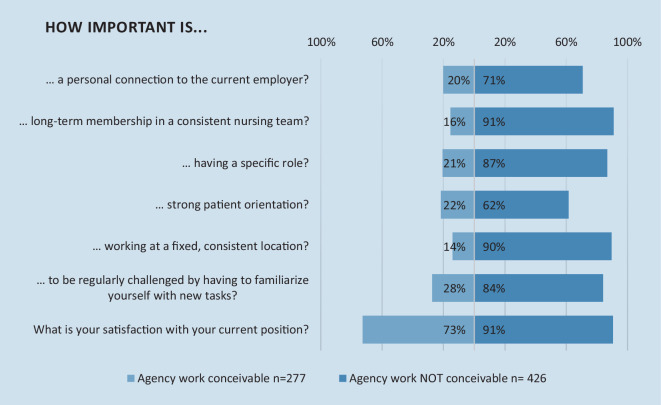
Importance of aspects of the job environment to nurses stratified by whether they consider agency work as conceivable for themselves

Regarding their own expectations and priorities, regular employees differed a lot based on whether they could conceive ever working in agency work (*n* = 277, 27%) or not (*n* = 426, 41%). Of the latter, 282 (91%) note that belonging to the same nursing team long-term was important or very important to them. On the other hand, of those who could conceive of doing agency work, only 55 (20%) saw this as a priority. The situation looks similarly for a fixed place of work (Fig. 3).

**Fig. 4:**
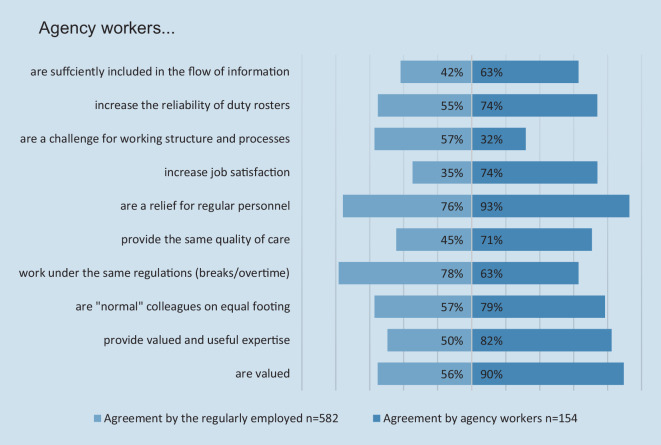
Assessment of agency workers by the regularly employed and agency workers themselves

The views of regularly employed and agency worker nurses on the assessment of agency work were assessed separately and compared (Fig. 4). Whereas 90% of the 154 agency workers agreed or completely agreed that agency workers were being valued, only 56% of the 582 regularly employed nurses shared that opinion about agency workers. Regarding legal requirements for breaks, off-time, and work hours, 78% of regularly employed participants vs. 63% of agency workers agreed that the same rules applied for both groups.

While almost all (93%) of agency workers believe their contributions make work easier for regular personnel, only 76% of traditional employees agree with that assessment. On the other hand, more than half of regular employees consider the deployment of agency workers as an additional challenge for operational procedures, whereas only a third of agency workers share that impression about their effect. Similarly, while 74% of agency workers agreed or completely agreed that their deployment allows for a more reliable duty roster, only 55% of regular employees shared these assessments. These discrepancies are mirrored in the overall assessment on the effect of agency worker deployment on job satisfaction: With 75% of agency workers believing their deployment to improve job satisfaction on the ward, only a third of regular employees feel that way. In general, agency workers seem to perceive their deployment as valuable.

**Fig. 5:**
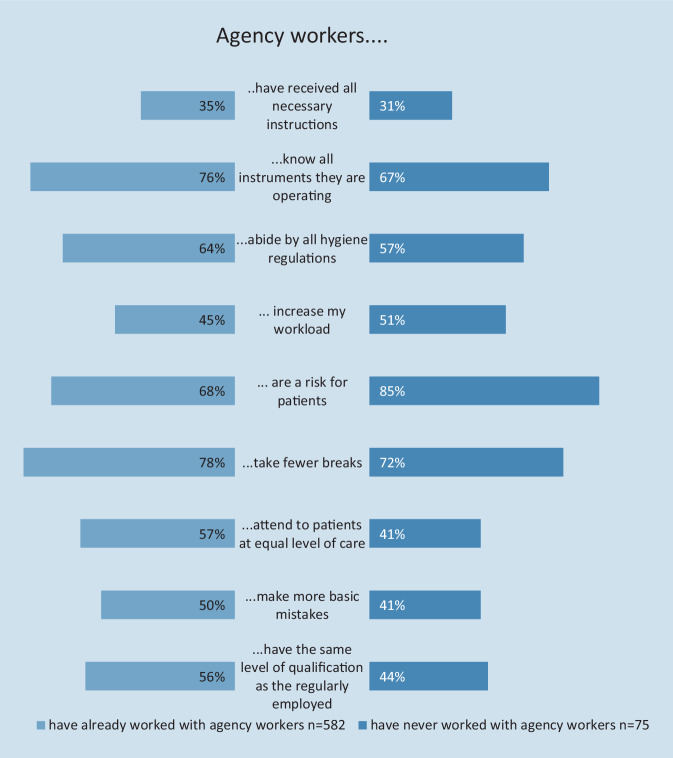
Views on agency workers by the regularly employed, stratified by whether they have experience working with agency workers

To further differentiate the assessment of the effect of the deployment of agency workers, regular employees were stratified by whether they had already worked with agency workers. Several aspects were to be graded with up to five stars. Of the points listed, none received four or five stars by any of the participants. Markedly, the question whether agency workers had received all the necessary instructions and onboarding was graded on average with 2.4 of five stars. For other aspects, assessments differed between those participants who had experience working with agency workers (*n* = 582) and those who never had worked with agency workers (*n* = 75; Fig. 5). Of those with no experience working with agency workers, a majority (*n* = 63, 85%) were convinced that agency workers are a danger for patients. Of those with experience, 334 (68%) shared that view. That agency workers were able to provide an equal quality of care was only confirmed by 334 (57%) of participants with and 30 (41%) of participants without experience working with agency workers.

## Discussion

To understand the motivations and job satisfaction factors of intensive and intermediate care nurses as well as their perception of working conditions, an electronic survey was conducted among 1203 participants in the German-speaking region using primarily snowball sampling. Of special interest was the opinion of and about agency workers in that field and what factors motivate them to decide on one form of employment over another.

Interestingly, it seems that neither reimbursement nor the form of employment alone seem to be a sufficient explanatory factor for job satisfaction, as regular employees with a side job in agency work seem to experience the worst overall job satisfaction. It thus cannot be said that they are able to enjoy “the best of both worlds”. Of course, the very fact that they take on a side job suggests that their primary employment does not fulfill all their needs.

In terms of the outlook on agency work in general, while certainly different perspectives were to be expected, the extent is interesting: Whereas agency workers, expectedly, consider their contributions beneficial, standard employees are far from convinced—to the point where a significant portion of that population considers agency workers a danger to the patient. Given the previous results suggesting a lack of evidence for differences in patient outcome as well as the fact that these views seem to be somewhat attenuated by having actual experience working with agency workers suggest that there is a certain level of distrust of the unknown involved. One very real factor, on which even agency workers agree, however, is that they often to not receive the instructions needed to use the equipment they need in their work. It is beyond the scope of this study to assess whether this is because of an implicit assumption that such instructions have been provided by the agency or simple oversight. It is, of course, a problem that should be addressed, even if the previous results on patient outcomes suggest that the issues arising from lacking instructions may well be compensated by colleagues. The compensation effort directly affects the workload of and the respect from regularly employed colleagues and thus directly impacts job satisfaction. To what extent is likely to be a factor of the overall team composition and qualifications, with ICUs with a better nurse/patient ratio having a greater ability to compensate individual shortcomings—but this can only be seen as a trade-off for the potential at higher job satisfaction the nurse/patient ratio offers at the same time. But the failure to provide the instructions required by the Medical Devices Directive at a minimum suggest the possibility of a very real risk for patients, and certainly a significant liability risks for all involved—including the employer. The fact that agency workers often report not being expected to attend meetings begs the question whether stronger integration into teams may help alleviate both information gaps and distrust.

Another strong difference between agency workers or regular employees who could conceive doing agency work, and other regular employees was their preferences regarding team and task diversity. Whereas traditional regular employees were interested in long-term membership in a single team, agency workers and those considering agency work were interested in experiencing a variety of different environments and tasks. This is particularly striking since the actual tasks to which agency workers are usually assigned are of a very limited spectrum.

As always, there are some limitations to this study: As there is no systematic record of nurses in intensive care, it was neither possible to calculate an adequate sample size in advance nor to truly conclude from the sample polled to the statistical population as such. Due to the use of snowball sampling, it is not even possible to calculate an actual return rate. This kind of survey always has a risk of bias as only certain subgroups feeling motivated to reply, e.g., certain age groups being more or less open to online surveys. That being said, the authors believe that the use of a broad spectrum of channels to approach participants alleviates this risk of bias to some degree. In addition, this kind of survey always has a risk of response bias in the form of acquiescence bias, central tendency bias, social desirability or other forms [2, 14]. To counter these issues, a variety of steps have been taken—not only were the questions randomized, but the orientation of the items in the modified Likert-scale were regularly switched. Furthermore, questions were not posed to ask for agreement, but opinion, and to minimize central tendency bias, items such as “don’t know” or “undecided” were omitted—participants had to decide one way or other. These points and the option to add free text to explain their position beyond strict scaled items for various points reduce the risk of bias.

All in all, job satisfaction is significantly skewed towards the negative for all groups. To change that, and to motivate agency workers to return to traditional forms of employment, more than financial factors need to be considered. A set nurse to patient ratio is seen as key by all groups, and other studies suggest that the target should be two patients per nurse throughout all shifts and all days [9]. If re-entry of agency workers into the regular workforce is seen as a goal, care also has to be taken to take into account their distinct outlook on the desirable task spectrum, with those active or interested in agency work preferring a more diverse and flexible spectrum and pure traditional employees preferring to be rooted in a fixed team.

At the moment, it may well be considered a form of “career” that the dissatisfaction in regular employment leads to seeking escape first in a side job or higher compensation to then either fully move into agency work or out of nursing altogether. This is supported by the fact that in the compensation part of this study, the wish to go into agency work was directly correlated with compensation: the higher the personal net income was, the lower this desire [4].

On the other hand, this means that one certain aspect of agency work on care in intermediate and intensive care units is a financial one: With a low supply of trained nurses and a high demand, the price for agency work is substantially higher than for regular employees—though not just due to this leverage but also the need for intermediaries in the agencies who need to be compensated.

At the same time, many of those who do work via agencies have an individual interest in certain aspects of that work—especially diversity of tasks and regular new challenges. They will try to fulfill those interests in any legal framework. A discussed ban on agency work is thus unlikely to be helpful. If it is seen as a desirable political goal to reduce agency work, three factors which motivate nurses to leave traditional models of employment need to be considered: Compensation—as discussed elsewhere [4], a personal net income of €3200 is considered sufficient and appropriate—work conditions and a specific improvement in appreciation by direct supervisors manifest in individual career development perspectives and duty rosters taking better into account both the legal requirements, onboarding necessities as well as the individual interests in specific work environments and task spectra. First and foremost, however, duty rosters need to become reliable, to ensure an adequate work–life balance.

Seeing nurses as a mere adjustable variable that can be deployed as needed as a stop-gap measure regardless of individual interests and aptitudes is at best a temporary solution with long-term negative effects on both job satisfaction and patient care. Three factors have been shown to influence patient outcome: the patient to nurse ratio, the qualification of the nurses caring for the patients, and their job satisfaction [5]. The latter may well be a factor in driving nurses towards agency work due to better pay and/or more flexible work conditions. In this context, it is particularly disturbing that agency workers regularly do not receive on-site instructions into the use of the equipment to be used. It is evident that no agency could possibly train agency workers on any and all instruments and instrument combinations they might encounter at all potential deployment sites. As such, this is a very manifest risk for the quality of care, but also a violation of legal requirements.

Further studies are needed to understand, what the critical ratio of regular to agency worker nurses is and what influence it has on the patients. Directly comparing factors such as skills in delirium recognition or rate of early mobilization could help in that effort.

This study has shown that a lot of intensive and intermediate care nurses bring a high degree of experience and motivation to their job which probably helps to compensate a lot of other risks for patient care. But both demographic change and job motivation are leading to a steady exodus, jeopardizing this buffer capability. To curb this development, a more systematic record of intensive and intermediate care units and their personnel in order to develop specific recommendations. The appropriate quality standards should, ideally, be defined by the intensive care nurses themselves.

## Practical conclusion

- Agency workers and regular employees have substantially different views on the effects of agency workers on the work environment and patient care.
- Agency workers and regular employees have distinct interests and expectations on their work, with agency workers preferring diversity and new challenges over consistency and a “home” team.
- Agency workers are often left out of the information flow, up to and including legally required instructions.
- Job satisfaction is low among all groups, but probably lowest among regular employees with a side job.
- Side jobs and agency work can be seen as escape attempts to compensate lack of job satisfaction with increased compensation.
- Compensation, reliability of duty rosters, reliable patient to nurse ratios and respect and appreciation by superiors are key factors that need to be addressed to curb the exodus from care.

## Declarations

For this article no studies with human participants or animals were performed by any of the authors. All studies mentioned were in accordance with the ethical standards indicated in each case.
