# Erratum to: Influence of working conditions and salary on agency work for intermediate and intensive care units

**DOI:** 10.1007/s00063-023-01020-z

**Published:** 2023-05-25

**Authors:** C. Hermes, U. Gaidys, K. Blanck-Köster, E. Rost, C. Petersen-Ewert

**Affiliations:** 1 Hochschule für Angewandte Wissenschaften Hamburg (HAW Hamburg), Alexanderstr. 1, 20099 Hamburg, Germany; 2 IU Internationale Hochschule GmbH, IU University of Applied Sciences, Juri-Gagarin-Ring 152, 99084 Erfurt, Germany; 3 Fakultät Wirtschaft und Soziales—Department Pflege and Management, Hochschule für angewandte Wissenschaften Hamburg, Hamburg, Germany

**Erratum to:**

**Med Klin Intensivmed Notfmed 2022**

10.1007/s00063-022-00969-7

In diesem Artikel waren die Adressdaten bzw. die Daten zur institutionellen Zugehörigkeit für den Autor E. Rost falsch angegeben. Statt Fakultät Wirtschaft und Soziales—Department Pflege and Management, Hochschule für angewandte Wissenschaften Hamburg, Hamburg, Germany hätte sie IU Internationale Hochschule GmbH, IU University of Applied Sciences, Erfurt, Germany lauten sollen. Der Originalbeitrag wurde korrigiert.

